# c-Myc dependent expression of pro-apoptotic Bim renders HER2-overexpressing breast cancer cells dependent on anti-apoptotic Mcl-1

**DOI:** 10.1186/1476-4598-10-110

**Published:** 2011-09-07

**Authors:** Mario Campone, Bélinda Noël, Cécile Couriaud, Morgan Grau, Yannis Guillemin, Fabien Gautier, Wilfried Gouraud, Catherine Charbonnel, Loïc Campion, Pascal Jézéquel, Frédérique Braun, Benjamin Barré, Olivier Coqueret, Sophie Barillé-Nion, Philippe Juin

**Affiliations:** 1 Centre de Recherche en Cancérologie Nantes-Angers - UMR 892 - INSERM/Université de Nantes, Institut de Recherche Thérapeutique de l'Université de Nantes 8 Quai Moncousu BP 7072144007 Nantes Cedex 1 France; 2 Service d'Oncologie Médicale, Institut de Cancérologie de l'Ouest, CLCC René Gauducheau, Bd J. Monod, 44805 Nantes - Saint Herblain France; 3 Unité de Biostatistique et de Biologie Intégrée, Institut de Cancérologie de l'Ouest, CLCC René Gauducheau, Bd J. Monod, 44805 Nantes - Saint Herblain France; 4 Unité Mixte de Génomique du Cancer, Hôpital Laënnec Bd J. Monod, 44805 Nantes - Saint Herblain Cedex France; 5 Département de Biologie Oncologique, Institut de Cancérologie de l'Ouest, CLCC René Gauducheau, Bd J. Monod, 44805 Nantes - Saint Herblain France; 6 Institut de Cancérologie de l'Ouest, CLCC Paul Papin, 2 rue Moll, 49033 ANGERS France

## Abstract

**Background:**

Anti-apoptotic signals induced downstream of HER2 are known to contribute to the resistance to current treatments of breast cancer cells that overexpress this member of the EGFR family. Whether or not some of these signals are also involved in tumor maintenance by counteracting constitutive death signals is much less understood. To address this, we investigated what role anti- and pro-apoptotic Bcl-2 family members, key regulators of cancer cell survival, might play in the viability of HER2 overexpressing breast cancer cells.

**Methods:**

We used cell lines as an *in vitro *model of HER2-overexpressing cells in order to evaluate how anti-apoptotic Bcl-2, Bcl-xL and Mcl-1, and pro-apoptotic Puma and Bim impact on their survival, and to investigate how the constitutive expression of these proteins is regulated. Expression of the proteins of interest was confirmed using lysates from HER2-overexpressing tumors and through analysis of publicly available RNA expression data.

**Results:**

We show that the depletion of Mcl-1 is sufficient to induce apoptosis in HER2-overexpressing breast cancer cells. This Mcl-1 dependence is due to Bim expression and it directly results from oncogenic signaling, as depletion of the oncoprotein c-Myc, which occupies regions of the Bim promoter as evaluated in ChIP assays, decreases Bim levels and mitigates Mcl-1 dependence. Consistently, a reduction of c-Myc expression by inhibition of mTORC1 activity abrogates occupancy of the Bim promoter by c-Myc, decreases Bim expression and promotes tolerance to Mcl-1 depletion. Western blot analysis confirms that naïve HER2-overexpressing tumors constitutively express detectable levels of Mcl-1 and Bim, while expression data hint on enrichment for Mcl-1 transcripts in these tumors.

**Conclusions:**

This work establishes that, in HER2-overexpressing tumors, it is necessary, and maybe sufficient, to therapeutically impact on the Mcl-1/Bim balance for efficient induction of cancer cell death.

## Background

Breast cancer is a heterogeneous disease, composed of distinct entities with differing underlying pathogenic processes. One such entity is the so-called HER2 subtype, which is characterized by amplification and/or overexpression of this member of the human epidermal growth factor receptor (HER) family. HER2 is an orphan receptor with intrinsic tyrosine kinase activity [1] whose activation results from the dynamic heterodimerization of HER receptors members [2]. This activates a large repertoire of transforming signaling molecules and pathways that are, to a great extent, shared by HER members.

Excess HER2 signaling leads to numerous oncogenic processes, including cell proliferation and survival [1]. The major signaling pathways activated by HER2 include the RAS-Raf1-Mek-Erk and the PI3K-Akt pathways. Akt signaling leads to mTOR activation. The mTOR signaling complex 1 (mTORC1) helps maintaining protein synthesis through phosphorylation of at least two direct targets, eukaryotic initiation factor (eIF) 4E-binding proteins (4E-BPs) and ribosomal protein S6 kinases (S6Ks) [3] that regulate the activity of EIF4F, a heterotrimeric complex required for the cap-dependent ribosome recruitment phase of translation initiation.

Activation of the Ras-MAPK-Erk and PI3K-Akt-mTOR pathways both culminate in activation of transcriptional programs, as well as cyclin dependant kinases, that lead to progression through the cell cycle. Current evidence indicates that, through either of these pathways, HER2 signaling can regulate c-Myc, a multifunctional transcription factor involved in cell cycle progression (see [4] and references therein). In particular, mTORC1 activity might contribute to cell cycle progression in HER2 overexpressing cells, as c-Myc expression is critically dependent upon EIF4F activity in cells with high Akt activity [5,6]. Consistent with this, inhibition of mTORC1 by RAD001 (everolimus) potently inhibits cell cycle progression of HER2 overexpressing breast cancer cells [7].

In addition to their deregulated proliferation, HER2 overexpressing cells exhibit altered survival signals. Breast cancer cells overexpressing HER2 are resistant to an array of cytotoxic agents and radiation damage [8,9]. In particular, anti-apoptotic signals associated with alterations of the downstream Ras-MAPK-Erk and PI3K-Akt-mTOR pathways contribute to chemo- and radioresistance. If targeting these survival signals is expected to be of therapeutic benefit in combination with cytotoxic approaches, a well-designed inhibition of some of these survival signals could have a more radical effect and directly promote tumor destruction. Indeed, some of the survival signals harbored by HER2 overexpressing cells might directly contribute to cancer progression by allowing cancer cells to survive to constitutive death signals. The existence of such signals is suggested, at least in part, by the fact that the kinase cascade triggered by the hyperactivity of receptors of the HER family can be "addictive" to cancer cells [10]. Such apparent addiction seems to result from the fact that hyperactivity of HER pathways has tumor promoting (survival) effects, but also tumor suppressive (death promoting) ones [11,12]. Death signals downstream of EGFR signaling have been reported, but not fully described in molecular details [10]. Moreover, it has remained unknown whether similar signals are initiated downstream of HER2. Investigating whether constitutive death and compensatory survival signals exist in HER2 overexpressing cells is of importance, as it may lead to the identification of a critical event in the HER2 network that needs to be altered by current targeted therapies, or that could be directly targeted without altering the rest of the network with great therapeutic benefit.

An investigation of the roles played by the Bcl-2 family of proteins in the survival of HER2 overexpressing cells may prove very useful to address this issue. This family of interacting proteins represents an integrating node towards which converge numerous death and survival signals in mammalian cells, including these induced by oncogenic signals [13]. Anti-apoptotic Bcl-2 homologues preserve mitochondrial integrity by opposing the activity of multi-domain pro-apoptotic Bcl-2 family members Bax and Bak, which display sequence conservation throughout three Bcl-2 homology (BH) domains (BH1-3), and that of their upstream effectors, the BH3-only proteins (e.g. Bim, Puma, Bad...). This occurs essentially by physical interactions between anti- and pro-apoptotic members which allows the former to negatively control the activation, and the activity, of pro-apoptotic Bax and Bak (themselves essential actors of the apoptotic response of mammalian cells to multiple stimuli). Anti-apoptotic Bcl-2 homologues (e.g. Bcl-2, Bcl-xL, Mcl-1) control the sensitivity to conventional pro-apoptotic therapy of tumor cells. In certain instances, their expression is necessary to maintain the survival of cancer cells [14,15], indicating that they may be required to counteract constitutive death signals. There is substantial evidence that the balance between anti- and pro-apoptotic proteins of the Bcl-2 family is biased in favor of survival proteins during breast carcinogenesis. Most breast cancers arise from epithelial cells that express Bcl-2, Bcl-xL and Mcl-1 [16,17], and enhanced expression of these proteins is almost systematically found in transformed mammary epithelial cells. Signaling pathways downstream of HER2 have numerous anti-apoptotic effects on Bcl-2 family members [18-20].

In this study, we investigated whether and how the imbalance in favor of survival proteins of the Bcl-2 family, which is induced by the sustained activity of signaling pathways downstream of HER2, contributes to survival maintenance in HER2 overexpressing breast cancer cells. We herein demonstrate that such cells undergo apoptosis upon depletion of Mcl-1, and that this Mcl-1 dependence is due to their constitutive expression of the pro-apoptotic protein Bim. The latter expression is a direct consequence of oncogenic signaling, as it is due to mTORC1 dependent expression of c-Myc, which occupies regions within the Bim promoter.

## Methods

### Reagents, antibodies and siRNAs

The following primary antibodies were used for western blotting: anti-actin from Millipore (mouse, MAB1501R), anti-ß-tubulin from Sigma (mouse, T0198), anti-Bcl-xL antibody from Transduction Lab. (rabbit, 610212); anti-Bcl-2 from Dako (mouse, M0887), anti-Mcl-1 from Santa Cruz (rabbit, B0410), anti-Puma from Calbiochem (rabbit, PC686); anti-Bim from Chemicon International (rabbit, 17003), anti-c-Myc from Cell Signaling (rabbit, 9402), anti-Foxo3A from Upstate (rabbit, 07-702), anti-phospho-p70 S6 kinase (Thr 388) from Cell Signaling (mouse, 9206). The following primary antibodies were used in chromatin immunoprecipitation assays (ChIP): anti-c-Myc (sc-764), anti-E2F1 (sc-193) from Santa Cruz (USA). Horseradish peroxidase-conjugated antibodies and enhanced chemiluminescence reagents were obtained from Santa Cruz (USA). Novartis provided RAD001. Unless indicated, all other reagents used in this study were obtained from Sigma. The following siRNAs were used: si-control A from Santa Cruz (sc-37007), si-Bcl-2 from Santa Cruz (sc-29214), si-Bcl-xL from Dharmacon (L-003458-00), si-Mcl-1 from Ambion (120644), si-Bim from Cell Signaling (6461), si-Puma from Dharmacon (L-004380-00), si-Myc from Santa Cruz (sc-29226), si-Foxo3A from Invitrogen (FOXO3a Validated Stealth DuoPak)

#### Cell lines

BT474, SKBR3 and MCF7, obtained from ATCC, were grown at 37°C with 5% of CO2 and humidified atmosphere. BT474 and MCF7 cells were grown in RPMI-1640 medium supplemented with 10% FBS (Fetal Bovine Serum), 1% glucose, 0,1% insulin, 1% Na-pyruvate, 1% non essential amino acids, 5% peni streptomycin. SKBR3 were grown in Mc Coy's 5A medium supplemented with 10% FBS, 5% glutamine, 5% peni streptomycin. **The non-transformed mammary epithelial cell line MCF10A was obtained from ATCC and grown in the recommended culture medium**.

### Transient RNA interference and drug treatment

One day prior transfection, 2.10^5 ^cells/well were seeded in 6-well plates with complete medium. Cells were transfected with siRNA oligonucleotides using Lipofectamine™ RNAiMax (InVitrogen) according to the manufacturer instructions. Briefly, cells were gently washed with PBS (Phosphate Buffered Saline) before transfection with a mix containing OPTIMEM, transfection reagent and 60 pmol of siRNA. After 5 hours of incubation, cells were gently washed with PBS and fresh complete medium was added. When applicable, a second transfection was performed 24 hours later following the same protocol. Adherent and floating cells were collected 48 hours later to perform western blot analysis or cell death investigations. Treatment of BT474 cells with RAD001 (20 nM) was performed on cells seeded in 6-well plates at 2.10^5 ^cells/well the day before and analysis was performed as described above.

### Western-blot analysis

Cells treated with RAD001 and/or the indicated siRNAs were lysed as follows. Floating and adherent cells were washed twice with cold-PBS. They were then lysed in lysis buffer (1% SDS, 10 mM EDTA, 150 mM NaCl, 20 mM Tris-HCl, pH 8.1, 1 mM phenylmethylsulfonyl fluoride, 2 μg/ml leupeptin, 5 μg/ml aprotinin, 1 μg/ml pepstatinA, 0.5 M NaF, 100 mM Na3VO4) and extracts were sonicated six times for 15s each. Supernatants were recovered by centrifugation at 12000 rpm for 10 min at 4°C.

To obtain tumor lysates, tumor tissue samples were surgically collected from untreated patients and processed in two parts by a pathologist: the first part was fixed in 10% neutral buffered formalin for standard histological analysis and determination of the HER2 by immunohistochemistry, and the second part was immediately snap-frozen in liquid nitrogen and stored at -180°C. This second part was crushed in liquid nitrogen using a sterilized mortar. After three washes in PBS, the samples were resuspended in a comparable volume of lysis buffer and extracts were sonicated on ice for 15 minutes. Supernatants were recovered by centrifugation at 12000 rpm for 10 min at 4°C.

Lysates prepared as described above (70 μg of total proteins) were separated by SDS-PAGE (12% polyacrilamide gel) under reducing conditions followed by transfer to a 0.45 μm PVDF membrane (Immobilon-P transfer membrane, IPVH00010). Non-specific binding was blocked by one hour incubation at room temperature in TBS-T (Tris Buffered Saline Tween, 25 mM Tris, 150 mM NaCl, 0.05% Tween 20, pH = 8.0) containing 5% (v/v) of blocking reagent (Western blocking reagent, Roche, 11921681001). Primary monoclonal antibodies were incubated for one hour at 37°C. After 3 washes with TBS-T, membranes were incubated with peroxidase conjugated secondary antibody for one hour at 37°C. Following 3 washes with TBS-T, blots were revealed using the chemiluminescent blotting Substrate Kit (Roche).

### Cell death assays

Following the indicated treatments, cells were labeled with the IOTest anti-APO2.7-PE (Beckman Coulter, PN IM2088U) according to the manufacturer's instructions. Briefly, floating and adherent cells were washed once in PBS, transferred in 96 well plates and washed twice more in cold PBS. Cells were then resuspended in 500 μl of labeling mix diluted in PBS and incubated in the dark for 15 minutes at RT. Cells were then washed in PBS and either immediately analyzed by FACS or fixed in 1% paraformaldehide for delayed FACS analysis. APO2.7 positive cells were analyzed using the FL1 channel (Ex. 486-580 nm/Em. 568-590 nm) of a FACS CaliburTM cytofluorometer (BD). **Annexin V staining was performed similarly, according to the manufacturer's instructions**.

### Mammosphere assays

BT474 cells treated with the indicated siRNA were plated as single cells in ultra low attachment plates (Corning) at low density (500 viable cells/cm^2^). They were grown in serum-free mammary epithelial cell growth medium containing DMEM-F12 (Sigma) supplemented with B27 (Gibco) and MEGM singlequots (Lonza France), as previously described [21]. Mammosphere-forming unit were counted as number of mammospheres ≥50 mm.

### Chromatin Immunoprecipitation (ChIP) assays

BT474 cells treated or not with RAD001 (20 nM) were washed and cross-linked with formaldehyde at room temperature for 8 min essentially as previously described [22]. Reaction was stopped with 10 ml of 125 mM glycin solution. Cells were washed with cold PBS and lysed in 500 μl of lysis buffer (1% SDS, 10 mM EDTA, 50 mM Tris-HCl pH 8.1, 1 mM PMSF, 5 mM NaF, 5 mM Na3VO4, 2 μg/ml leupeptin, 5 μg/ml aprotinin, 1 μg/ml pepstatin), and sonicated five times for 20 seconds each. Supernatants were then recovered by centrifugation at 12 000 rpm for 10 min at 4°C, diluted once in dilution buffer (1% Triton X-100, 2 mM EDTA, 150 mM NaCl, 20 mM Tris-HCl pH 8.1) and subjected to one round of immunoclearing for 2 h at 4°C with 2 μg of sheared salmon-sperm DNA, and 20 μl of proteinG-agarose coated with salmon sperm DNA (Millipore) (of 50% slurry). Immunoprecipitation was performed overnight with specific antibodies and IgG control, and then 2 μg of sheared salmon-sperm DNA and 20 μl of proteinG-agarose coated with salmon sperm DNA (Millipore) (of 50% slurry) were further added for 1 h at 4°C. Note that immunoprecipitations were performed in the presence of 1% Igepal CA-630. Immunoprecipitates were washed sequentially for 10 min each in TSE I (0.1% SDS, 1% Triton X-100, 2 mM EDTA, 20 mM Tris-HCl pH 8.1, 150 mM NaCl), TSE II (0.1% SDS, 1% Triton X-100, 2 mM EDTA, 20 mM Tris-HCl pH 8.1, 500 mM NaCl), and TSE III (250 mM LiCl, 1% NP-40, 1% deoxycholate, 1 mM EDTA, 10 mM Tris-HCl pH 8.1). Beads precipitates were then washed once with TE buffer and eluted once with 1% SDS, 100 mM NaHCO3. Eluates were heated at 65°C for 6 hours to reverse the formaldehyde cross-linking. DNA was precipitated using classical procedures. Real-time PCR was used for ChIP analysis and quantification. The ChIP has been calculated as binding to region of interest/IgG control, divided by binding to negative control region/IgG control. The following primers were used:

region -61/+50 of the Bim promoter: Forward 5'-CAGTGATTGGGCGTAGGAG-3', Reverse 5'-ACGCAGAGCTCCAACAAACT-3'; Control region -1701/-1399 of the Aurora A promoter For 5'-ACTCCAGATCCCTCAGCTTAACCA-3' Rev 5'-CAAGTTATGGGACGGTGAACG-3'. All statistical analysis has been performed with Graphpad Software.

### Patient samples

As required by the French Committee for the Protection of Human Subjects, informed consent was obtained from study patients to use their surgical specimens and clinicopathological data for research purposes, and the local ethic committee approved protocols.

### Statistical analysis of published expression data

The impact of HER2 status **(as evaluated by immunohistochemistry) **on the expression of 20 genes of the Bcl-2 family was evaluated by means of Wilcoxon test. When the evaluation was performed in a "probe-matching" way, 2 pooled published cohorts for which Affymetrix data were available [23,24] were used after their conversion to a common scale. In a "gene-matching approach" the evaluation was performed on a larger pool obtained by merging 5 genomic published cohorts [23-27]. If multiple probes corresponded to a same gene, the median of probes was taken.

## Results

### Mcl-1 is highly expressed in HER2 overexpressing cancers, and is required to maintain the survival of HER2 overexpressing cells in vitro

**Figure 1:**
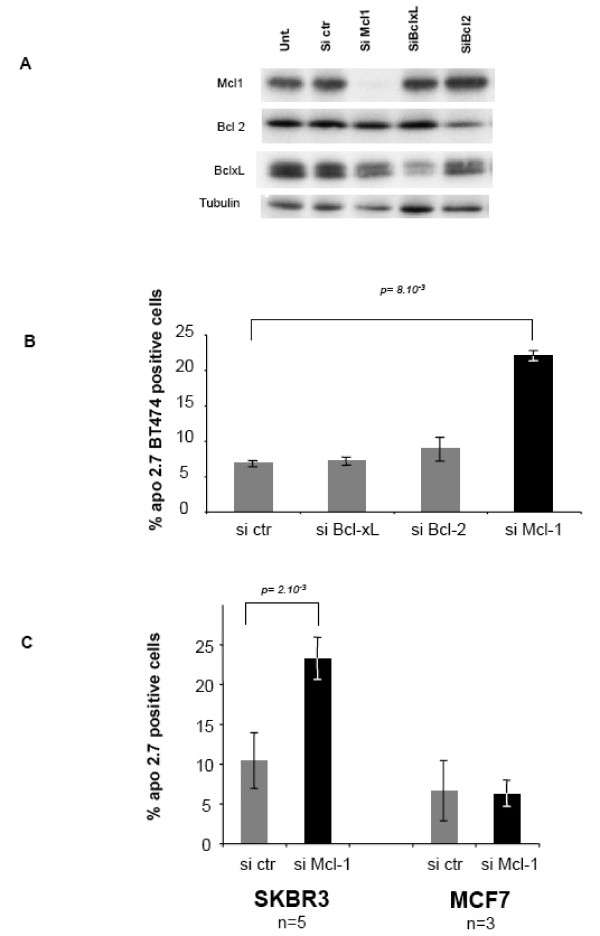
**RNA interference mediated knock-down of Mcl-1 induces cell death in HER2 overexpressing cells**. BT474 cells were left untreated or transfected with control siRNA, Bcl-xL siRNA, Bcl-2 siRNA or Mcl-1 siRNA as indicated. (A) Down regulation of the targeted proteins 48 hours after transfection was confirmed by western blot analysis, using tubulin as a loading control. (B) Forty eight hours after transfection, apoptosis was assessed by Apo 2.7 staining and FACS analysis as described in Materials and Methods. Data represented (% of Apo2.7 positive cells) are the means +/- standard errors of three independent experiments. p-values were assessed using a Student's t test. (C) Experiments were performed as described in B, using HER2 overexpressing SKBR3 cells, and ER expressing MCF7 cells, as indicated. Data are mean ± se of the indicated number of independent experiments. p-values were assessed using a Student's t test

The HER2 amplified BT474 breast cancer express detectable levels of the main anti-apoptotic Bcl-2 homologues Bcl-xL, Bcl-2 and Mcl-1 (Figure 1A). We investigated whether any of these proteins play a crucial role in maintaining the viability of BT474 cells in vitro using a RNA interference approach based on the transfection of small interfering RNAs (siRNA) targeting Bcl-xL, Bcl-2 or Mcl-1. **Transfection with control siRNA did not impact on the expression of these proteins compared to that found in non-transfected cells **(Figure 1A). **In contrast, transfection of BT474 cells with the targeted siRNA led to the selective down regulation of the targeted proteins 48 hours after treatment**. We analyzed the consequence of Bcl-xL, Bcl-2 and Mcl-1 depletion, under these conditions, on the viability of BT474 cells. We measured the expression, by the transfected cells, of the APO2.7 antigen, whose expression is restricted to dying, apoptotic cells. As shown in Figure 1B, knock down of Mcl-1 expression by RNA interference lead to the induction of apoptosis in a substantial fraction of cells. In contrast, depletion of either Bcl-xL or Bcl-2 did not induce apoptosis in BT474 cells. **Induction of cell death, and of apoptosis, by Mcl-1 depletion in BT474 cells was also confirmed by a trypan blue staining procedure and by Annexin V staining followed by flow cytometry analysis **(Additional File 1). Thus, Mcl-1 is specifically involved in preventing BT474 cells from spontaneously undergoing apoptosis.

Interestingly, we found that this feature of "Mcl-1 dependence" was displayed by another HER2 overexpressing cell line, SKBR3, as transfection with Mcl-1 siRNA was sufficient to induce rates of apoptosis in these cells also (Figure 1C and Additional File 2). In contrast, transfection with Mcl-1 siRNA, under the same conditions, had no detectable effect on the viability of ER positive MCF7 cells, that do not overexpress HER2 (Figure 1C) **despite down regulation of Mcl-1 (**Additional File 2**)**. **Notably, expression levels of Mcl-1 in the three cell lines was high compared to that found in the non transformed mammary epithelial cell line MCF10A (**Additional File 3**), indicating that signaling pathways leading to enhanced expression of Mcl-1 are active in transformed mammary epithelial cells, and in HER2 overexpressing ones in particular**.

**Figure 2:**
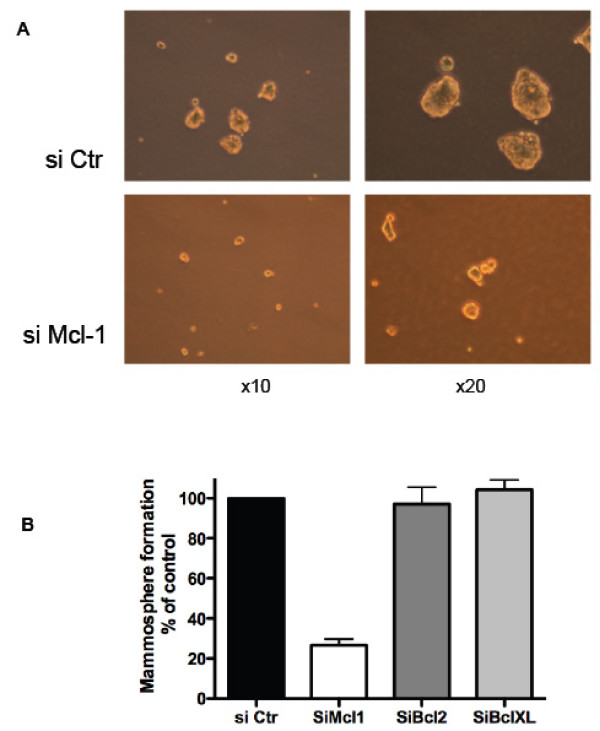
**Mcl-1 knockdown negatively impacts on mammosphere formation by BT474 cells**. BT474 cells were transfected with control or **the indicated **siRNA, as described in Figure 1. One day later, the resulting cells were plated in non-adherent conditions under mammosphere medium, as described in Methods. Mammosphere formation was analyzed by microscopy for (A) and the number of mammospheres formed per dish was enumerated (B) two days later. Data are mean ± se of 3 independent experiments normalized to data obtained with transfection with a control siRNA.

Transformed mammary epithelial cells, including established breast cancer cell lines such as BT474 cells [28], exhibit an inherent phenotypic plasticity and harbor a subpopulation of cells with features of cancer initiating cells (CIC). The latter cells, which are characterized by numerous parameters, including their ability to form spherical colonies in non-adherent culture conditions (mammospheres), were frequently described as being resistant to cell death induction by numerous stimuli [29]. This suggests that they may rely on survival signals distinct from these that are critical for the rest of the population. We thus investigated whether the Mcl-1 dependence of BT474 cells revealed above (in assays performed on the bulk population) applies to the subpopulation of CICs. To test this, we reasoned that, if BT474 CICs are Mcl-1 dependent, then a diminished ability to form mammospheres should be observed in a population of BT474 that has been depleted in Mcl-1. **The ability of BT474 cells to form mammospheres after transfection with siRNAs was thus evaluated**. As shown in Figure 2, the ability of Mcl-1 depleted BT474 cells to form mammospheres was significantly decreased compared to that of the same cells treated with a control siRNA. **In contrast, Bcl-xL or Bcl-2 knock down was insufficient by itself to affect mammosphere formation by BT474 cells (**Figure 2**)**. Taken together, these data indicate that the HER2 overexpressing BT474 cells require Mcl-1 to survive *in vitro*, and that this Mcl-1 dependence extends to their subpopulation of CICs.

To investigate whether pathways driving Mcl-1 expression are specifically active in HER2 overexpressing cancers, compared to other breast cancers, we analyzed the expression of 20 pro- and anti-apoptotic Bcl-2 family members from published gene-expression profiles of breast cancer patients. We based this analysis on studies in which the HER2 status of each tumor was available **and had been evaluated by immunohistochemistry, and that were performed using Affymetrix microarrays**. Two studies corresponded to these criteria, allowing to investigate expression profiles of 41 HER2 overexpressing tumors and 170 HER2 - ones (Table 1). Our evaluation was performed in a "probe-matching" way, using the 2 pooled aforementioned cohorts. Regarding the expression of anti-apoptotic genes (pro-apoptotic ones are evoked below), this evaluation revealed a statistically significant enrichment **(that is, a statistically significant higher expression**), in HER2 overexpressing breast tumors compared to other breast tumors, in one MCL1 specific probe and also in one BCL2L1 (Bcl-xL) one (Table 1). In contrast, other breast tumors appeared statistically enriched for three BCL2 specific probes (Table 1). Interestingly, when the evaluation was performed on a larger pool obtained by merging the two previously described cohorts with 3 additional genomic published cohorts (see Materials and Methods), using a "gene-matching" approach, an enrichment in MCL1 expression in HER2 overexpressing tumors (455 HER2- versus 71 HER2+, p = 0,0156), and in BCL2 in the other ones (216 HER2- versus 52 HER2+, p = 0,0145) was also found. In contrast, enrichment in BCL2L1 (268 HER2- versus 55 HER2+,) was no longer found. These molecular profiling analyses are mostly consistent with the notion that mechanisms leading to Mcl-1 transcription and expression are highly active in HER2 overexpressing breast cancers.

**Table 1:** Impact of HER2 status on the expression of Bcl-2 family members in mammary tumors

Probe	Gene	pValue	Enrichment in HER2+
P_1861_at	**BAD**	0,0294	-

P_203728_at	**BAK1**	ns	ns

P_208478_s_at	**BAX**	ns	ns

P_211833_s_at	**BAX**	ns	ns

P_211692_s_at	**BBC3**	ns	ns

P_203685_at	BCL2	< 0.0001	-

P_207004_at	BCL2	0,0157	-

P_207005_s_at	BCL2	0,0311	-

P_203684_s_at	BCL2	ns	ns

P_205681_at	BCL2A1	ns	ns

P_212312_at	BCL2L1	0.0054	+

P_206665_s_at	**BCL2L1**	ns	ns

P_215037_s_at	**BCL2L1**	ns	ns

P_221320_at	**BCL2L10**	ns	ns

P_222343_at	**BCL2L11**	0.0061	+

P_208536_s_at	**BCL2L11**	ns	ns

P_217955_at	**BCL2L13**	ns	ns

P_221241_s_at	**BCL2L14**	ns	ns

P_209311_at	BCL2L2	ns	ns

P_211725_s_at	**BID**	0,0086	+

P_204493_at	**BID**	ns	ns

P_205780_at	**BIK**	0.016	ns

P_221454_at	**BOK**	ns	ns

P_206864_s_at	**HRK**	ns	ns

P_206865_at	**HRK**	ns	ns

P_214056_at	MCL1	0,0097	+

P_200796_s_at	MCL1	ns	ns

P_200797_s_at	MCL1	ns	ns

P_200798_x_at	MCL1	ns	ns

P_214057_at	MCL1	ns	ns

P_204285_s_at	**PMAIP1**	0,0005	-

P_204286_s_at	**PMAIP1**	< 0.0001	-

The impact of HER2 status **(evaluated by immunohistochemistry) **on the expression of 20 genes of the Bcl-2 family was evaluated as described in Methods. The cohort contained 41 HER2+ and 170 HER2- ones. + and - indicate an enrichment and an impoverishment, respectively, in the HER2 overexpressing cohort. P values were assessed by a Wilcoxon test. **Pro-apoptotic genes are shown in bold**.

### The Mcl-1 dependence of HER2 overexpressing BT474 cells is due to constitutive expression of pro-apoptotic Bim

We investigated the molecular basis of the signal(s) that render(s) Mcl-1 necessary for the viability of HER2-overexpressing cells. Bcl-2 homologues promote survival in great part by counteracting pro-apoptotic counterparts, Bax/Bak and their upstream effectors the BH3-only proteins. Some BH3-only proteins, such as Bid, BIM or PUMA interact with all known anti-apoptotic Bcl-2 members, and activate Bax/Bak directly. They are therefore good candidates as proteins that can initiate death signals that make anti-apoptotic proteins (in our case, Mcl-1) required for survival. This is particularly true for Bim and Puma, that activate Bax/Bak in their native form, whereas cleavage of Bid is required for it to exert its pro-apoptotic activity [30].

**Figure 3:**
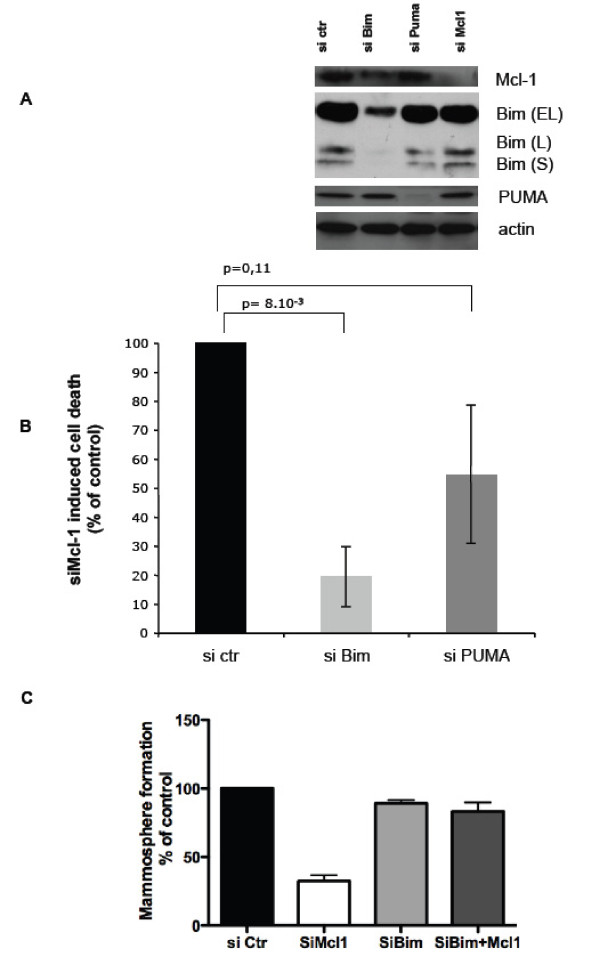
**Expression of pro-apoptotic Bim is required for induction of cell death and decrease in mammosphere formation by Mcl-1 knock down**. BT474 cells were transfected with control siRNA, Bim siRNA, PUMA siRNA and/or Mcl-1 siRNA as indicated. (A) Western blot analysis was performed to confirm the down regulation of the targeted proteins by each of the indicated siRNA. (B) Apoptosis was assessed 48 hours later by Apo 2.7 staining as described in Figure 1. Data are mean ± se of three independent experiments. They are expressed as a percentage of cell death obtained after Mcl-1 siRNA transfection of cells that had been transfected with control siRNA **(46% ± 5.2%)**. p-values were assessed using a Student's t test. (C) Mammosphere formation by BT474 cells transfected with the indicated siRNA was evaluated as described in Figure 2. Data are mean ± se of 3 independent experiments normalized to data obtained with transfection with a control siRNA.

We found that BT474 cells express detectable levels of Puma and of Bim whether cells were grown under control conditions or transfected with control, scramble siRNAs (Figure 3A and Additional File 4). **In contrast, these cells expressed barely detectable levels of Noxa, a BH3-only protein which functions as a selectiove inhibitor of Mcl-1 (**Additional File 4**)**. Regarding Bim, it has to be noted that we essentially detected its Bim Extra-Long (EL) form, whereas the Long (L) and Short (S) forms **were less expressed **in these cells (Figure 3). To investigate whether Bim or Puma play an active role in the Mcl-1 dependence of BT474 cells, these cells were transfected with control, Bim or Puma siRNA, which down regulated efficiently the targeted proteins (Figure 3A), prior to their transfection with Mcl-1 siRNA and investigation of cell death. Of note, neither Bim nor Puma siRNA affected cell viability by themselves **(**Additional File 1**and data not shown)**. Bim depletion robustly prevented cell death induced by transfection with Mcl-1 siRNA, **as measured by APO2.7 staining (**Figure 3B**) or by Annexin V staining (**Additional File 1**)**, indicating that this pro-apoptotic protein plays a major role in the Mcl-1 dependence of BT474 cells. In contrast, PUMA depletion had a much less pronounced and consistent effect on Mcl-1 knock down induced cell death **(**Figure 3A and Additional File 1**)**.

We investigated whether Bim contributes to the Mcl-1 dependence of the subpopulation of BT474 that are capable of forming mammospheres. Bim depletion had no impact in itself on mammosphere formation by BT474 cells. However, it abrogated the ability of Mcl-1 knock down to decrease the number of mammospheres formed by BT474 cells (Figure 3C). This is strong support to the notion that the Mcl-1 dependence of BT474 CICs also is due to Bim expression.

It rises from above that constitutive expression of Bim may contribute to render Mcl-1 necessary for the survival of HER2 overexpressing tumors. To analyze whether mechanisms leading to Bim transcription are particularly at stake in HER2 overexpressing tumors, we went back to our investigation of published gene-expression profiles of breast cancer patients using a probe-matching approach as described above. As shown in Table 1, we found a statistically significant enrichment, in HER2 overexpressing breast tumors compared to other breast tumors, in one BCL2L11 (Bim) specific probe. Regarding pro-apoptotic genes, a statistical enrichment in one BID specific probe and in one BIK specific probe was also found (Table 1). In contrast, other breast tumors appeared statistically enriched for two PMAIP1 (Noxa) specific probes and for one BAD specific one (Table 1). While this tends to suggest that pathways leading to Bim transcription might be more active in HER2 overexpressing breast cancers, this should nevertheless be taken cautiously. Indeed, we did not confirm a statistical enrichment for Bim expression in HER2 overexpressing cancers by our gene-matching approach involving 5 cohorts (described above), even though enrichment for BID and BIK and impoverishment for BAD and NOXA were confirmed.

**Figure 4:**
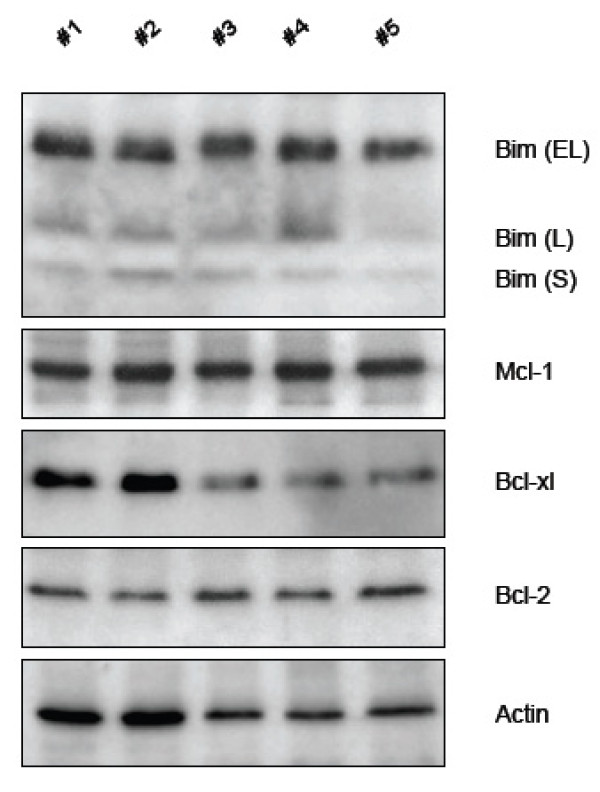
**HER2-amplified mammary tumors express detectable levels of Bim**. Tumor lysates were prepared and analyzed by western blotting as described in Materials and Methods.

In an independent attempt to confirm that HER2 overexpressing tumors express Bim, we prepared lysates from five tumors that had been diagnosed as HER2 overexpressing ones by immunohistochemistry and performed western blot analysis. As shown in Figure 4, these lysates expressed detectable levels of anti-apoptotic Bcl-2, Bcl-xL and Mcl-1. They also expressed detectable levels of Bim. Most importantly here, we picked these samples because they correspond to tumors that had received no treatment prior surgery. The expression of pro-apoptotic Bim detected does not, therefore, result from stress induced by treatment, but is more likely to result from signals that are inherent to the biology of these tumors.

### c-Myc contributes to Bim expression and Mcl-1 dependence of BT474 cells

**Figure 5:**
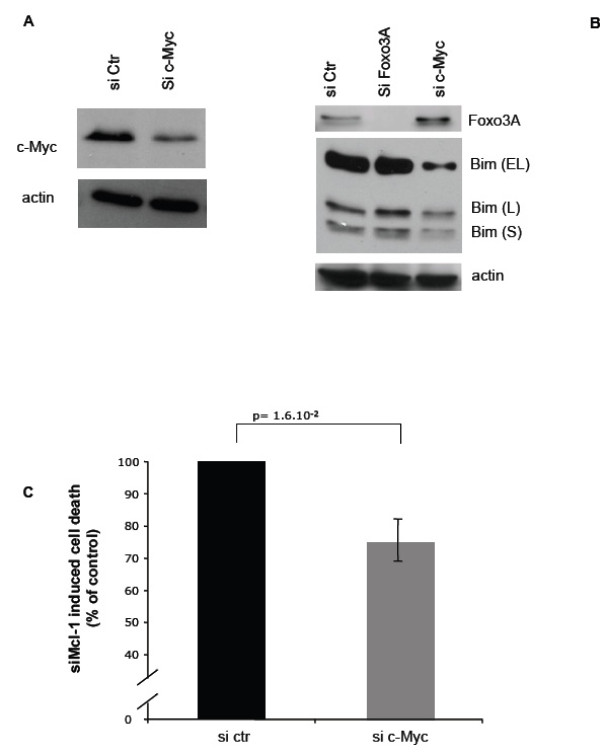
**c-Myc expression in BT474 cells impacts on Bim expression and Mcl-1 dependence**. BT474 cells were transfected with control siRNA, Foxo3A siRNA or c-Myc siRNA, as indicated. 48 hours later, western blot analysis was performed to confirm the effect of each siRNA on the expression of the targeted proteins and on Bim (EL), (L-) and (S), as indicated (A-B). In (C), BT474 cells were transfected with control siRNA, c-Myc siRNA, and/or Mcl-1 siRNA as indicated and apoptosis was assessed 48 hours later as described in Figure 1. Data are mean ± se of three independent experiments. They are expressed as a percentage of cell death obtained after Mcl-1 siRNA transfection of cells that had been transfected with control siRNA **(42.4% ± 6.8%)**. p-values were assessed using a Student's t test.

We investigated which signaling pathways might contribute to Bim expression in BT474 cells. Foxo3a is a member of the Foxo class of the forkhead family of winged helix transcription factors, which was reported to directly induce the transcription of Bim, in particular in some breast cancer cells [19]. However, a RNA interference approach that successfully down regulated Foxo3A expression in BT474 cells (Figure 5A) had no discernible effect on constitutive Bim protein expression (Figure 5B), ruling out that Foxo3A activity is responsible for this constitutive expression.

c-Myc is a transcription factor that resembles transcription factors of the basic helix-loop-helix leucine zipper (bHLH-LZ) family. It is a major regulator of cell proliferation but it is also capable of promoting apoptosis. In particular, it was reported to induce Bim in certain settings ([31] and see below). We used a RNA interference approach to specifically down-regulate c-Myc in BT474 cells (Figure 5A) and we found that it induced a significant decrease in the expression of Bim in the resulting cells (Figure 5B).

To investigate whether c-Myc is involved in the inherent Mcl-1 dependence of BT474 cells, these cells were transfected with control or c-Myc siRNA, prior to their transfection with Mcl-1 siRNA and investigation of cell death as described above. Of note, c-Myc siRNA had no impact on cell viability by itself (data not shown). As shown in Figure 5C, decreased c-Myc expression diminished cell death induced by transfection with Mcl-1 siRNA, indicating that this transcription factor contributes to the Mcl-1 dependence of BT474 cells.

### Decrease of c-Myc expression upon inhibition of mTORC1 diminishes Bim expression levels and mitigates the Mcl-1 dependence of BT474 cells

**Figure 6:**
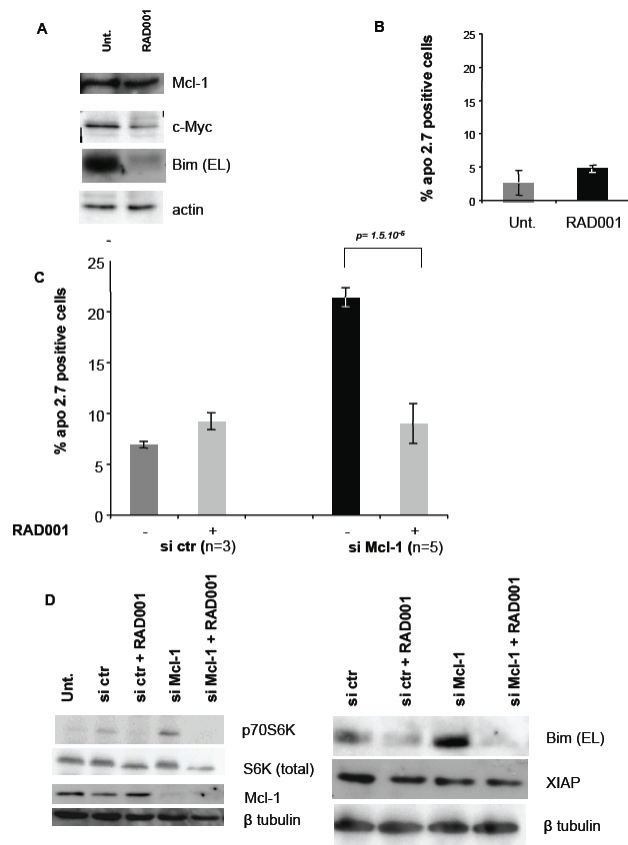
**Treatment of BT474 cells with the mTORC1 inhibitor RAD001 decreases Myc and Bim expression and mitigates Mcl-1 dependence**. (A-B) BT474 cells were treated with 20 nM RAD001 for 72 hours. (A) Western blot analysis was performed to document the effect of RAD001 treatment on Mcl-1, c-Myc and Bim expression. (B) Cell death was assessed by Apo 2.7 staining as described in Materials and Methods. Data are mean ± se of three independent experiments. p-values were assessed using a Student's t test. (C) BT474 cells were treated with 20 nM RAD001 or left untreated, as indicated. 24 hours later, cells were transfected with control or Mcl-1 siRNA and put back into control or RAD001 containing complete medium. Apoptosis was assessed 48 hours later by Apo 2.7 staining as described in Figure 1. Data are mean ± se of the indicated number of independent experiments. p-values were assessed using a Student's t test. (D) Western blot analysis was performed to document the effects of RAD001 treatment and of Mcl-1 siRNA on the phosphorylation of p70S6K and on Mcl-1, Bim and XIAP expression, using ß-tubulin as a loading control.

In HER2-overexpressing cells with high Akt activity, mTORC1 downstream of Akt is expected to actively contribute to c-Myc expression [6]. Thus, Bim expression in such cells may directly result from oncogenic signaling. To confirm this notion, we treated BT474 cells with the mTORC1 inhibitor RAD001, under conditions that proved sufficient to prevent their growth [7], arrest these cells in the G1 phase of the cell cycle (data not shown) and prevent phosphorylation of S6K (Figure 6D). Importantly, this treatment by itself did not induce significant apoptosis rates in BT474 cells (evaluated by APO2.7 staining as described above, Figure 6B) and had no detectable impact on Mcl-1 expression (Figure 6A). In contrast, this treatment lead to a decrease in c-Myc expression (Figure 6A). Coincidentally, RAD001 treatment significantly decreased Bim expression in BT474 cells (Figure 6A).

**Since c-Myc both affects Bim expression in BT474 cells as well as their Mcl-1 dependence, we then analyzed whether RAD001 treatment, which impacts on Bim expression, also impacts on such dependence**. Cells were treated with RAD001 or not prior to their transfection with control or Mcl-1 siRNA, and cell death rates were analyzed as described above. As shown in Figure 6C, RAD001 treatment did not enhance cell death rates induced by Mcl-1 siRNA, indicating that RAD001 has no pro-apoptotic effect even in Mcl-1 depleted BT474 cells. Instead, we found that RAD001 significantly prevented cell death induced by Mcl-1 siRNA. Western blot analysis (Figure 6D) showed that RAD001 treatment did not interfere with the ability of Mcl-1 siRNA to down regulate Mcl-1 and that, conversely, RAD001 treatment was still efficient (as judged by its effect on the phosphorylation of S6K) in Mcl-1 depleted cells. Moreover, RAD001 treatment decreased Bim expression in cells treated with a control siRNA and in Mcl-1 depleted cells (Figure 6D). In contrast, the expression levels of XIAP, another anti-apoptotic protein whose expression was reported to be enhanced by mTORC1 inhibition in some cases [32] were left unchanged by RAD001 treatment (Figure 6D). Thus, these data reveal a genuine anti-apoptotic effect exerted by RAD001 treatment in BT474 cells, which allows them to survive even when Mcl-1 is depleted and which correlates with a decrease in Bim expression.

### c-Myc occupies regions of the Bim promoter by an mTORC1 dependent process

**Figure 7:**
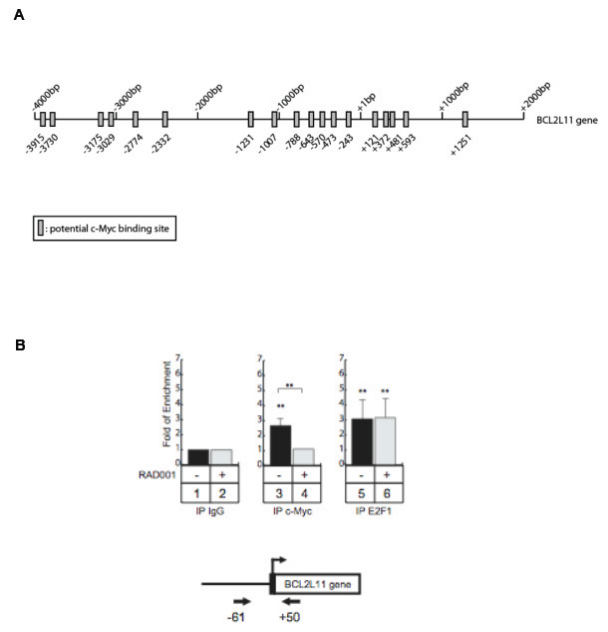
**Myc associates with the Bim promoter by a RAD001 dependent process in BT474 cells**. (A) Schematic representation of the potential Myc binding sites of the BCL2L11 promoter. (B) BT474 cells were treated **(2, 4, 6) **or not **(1, 3, 5) **with RAD001, soluble chromatin was immunoprecipitated with anti-Myc **(3, 4) **or anti-E2F-1 antibodies **(5, 6) **and DNA samples were then amplified using primers that cover the -61/+50 region of the BCL2L11 promoter. IgG immunoprecipitations were used as controls **(1, 2)**. Data are mean ± sd of 4 independent experiments normalized to data obtained with control IgG immunoprecipitations. p-values were assessed using a Student's t test.

In a last series of experiments, we analyzed whether the RAD001 sensitive, c-Myc dependent expression of Bim we detected in BT474 cells directly ensued from transcriptional regulation of Bim by c-Myc, *id est *from mTORC1 dependent occupancy of regions of the Bim promoter by this transcriptional factor. Using the UCSC genome browser (http://genome.ucsc.edu), we noticed that ChIP-on-chip experiments have already suggested that c-Myc can potentially bind to the BCL2L11 (Bim) promoter in HeLa cells. Moreover, Ouyang and collaborators have shown by ChIP-seq assays that c-Myc and its homologue N-Myc can be found associated with this gene in embryonic stem cells [33]. Consistent with these findings, transcription factor recognition site analysis of the BCL2L11 gene by Matinspector software http://www.genomatix.de showed the presence of a large number of potential c-Myc binding sites (Figure 7A).

To determine if c-Myc binds to the Bim promoter, we analyzed its recruitment by chromatin immunoprecipitation assays (ChIP) in BT474 cells. Results presented in Figure 7B show that c-Myc is recruited to the initiation transcription site of BCL2L11 gene. Of note, we found this to be associated with the binding of histone 3 (H3) acetylation (K9) and that of RNA polymerase II (data not shown), which is indicative of gene transcription. Interestingly, we also noticed the recruitment of the E2F1 transcription factor on this gene. Following mTORC1 inhibition by RAD001 treatment, as expected from the decrease of c-Myc expression under these conditions, an inhibition of c-Myc binding to the Bim promoter was observed (Figure 7B). This correlated with a loss of the transcription indicators (data not shown). In contrast, E2F1 binding was not affected following RAD001 treatment suggesting that RAD001-mediated inhibition of Bim expression is E2F1 independent.

Altogether, these data indicate that mTORC1 promotes Bim expression by stabilizing c-Myc on BCL2L11 promoter in the HER2-overexpressing breast cancer cell lines BT474.

## Discussion

We used, in this study, BT474 cells that overexpress HER2/neu, and in which signaling downstream of this member of the EGF receptor family is highly active. Our results establish that, despite the potent and numerous survival signals that are associated with HER2 activity, these cells rely on the expression of a single anti-apoptotic protein for their survival, as the down regulation of Mcl-1 is sufficient to induce significant rates of spontaneous apoptosis in these cells. Mcl-1 appears to be crucial even for the subpopulation of BT474 that have features of cancer initiating cells, as its depletion significantly reduces the number of mammospheres these cells can form. Since the co depletion of pro-apoptotic Bim (which reduces cell death rates induced by Mcl-1 depletion as discussed below) mitigates the effects of Mcl-1 knock down on mammosphere formation, these effects most likely result from the induction of cell death in sphere forming cells. We cannot formally rule out, however, that Mcl-1 contributes to the biology of cancer initiating cells by mechanisms other than regulation of cell survival *stricto sensu*. This aspect is currently being investigated in our laboratory.

Given the role played by Mcl-1 in maintaining the survival of HER2 expressing cells, and in maintaining a significant pool of cancer initating cells among them, pathways that lead to the expression of the anti-apoptotic protein Mcl-1 are expected to contribute to the pathogenesis of HER2 amplified mammary tumors. Conversely, pharmacological manipulations of these pathways may be of therapeutic benefit. Our investigation of published expression data hint on a selective enrichment for Mcl-1 trancripts in HER2 amplified mammary tumors compared to other mammary tumors. Thus, pathways that positively impact on the transcription of Mcl-1 may be particularly active in HER2 amplified tumors, either because they are directly triggered by this pathway or because their secondary activation contribute to the progression of this malignancy. One such pathway might be the one that relies on STAT3 activity which was shown to promote Mcl-1 transcription and to be activated in response to ligands that activate growth factor receptors with tyrosine kinase activity, including HER2 [34-36].

Mcl-1 protein and mRNA both have short half-lives. Mcl-1 mRNA has a G+C-rich 5'UTR and its translation is expected to be preferentially increased when the activity of EIF4F is elevated [37]. Our demonstration of a key role of Mcl-1 in the survival of HER2-amplified cells might thus have provided one rationale for the use of the mTORC1 inhibitor RAD001 against this malignancy. Our results nevertheless show that an impact of RAD001 on the viability of HER2-amplified cells, via an effect on Mcl-1 expression, may not be guaranteed. Concentrations of RAD001 that are sufficient to inhibit the growth and cell cycle progression of BT474 cells are indeed inefficient at inducing apoptosis and at down regulating Mcl-1 expression. The reason why inhibition of mTORC1, in conditions in which it is sufficient to promote cell cycle arrest and the down regulation of proteins involved in cell cycle control (such as c-Myc), does not affect Mcl-1 expression, is currently unclear. One possibility is that RAD001, like rapamycin, only partially inhibits mTORC1, affecting phosphorylation of rpS6 but leaving phosphorylation of 4EBP1 relatively unaltered. Increases in Mcl-1 protein levels downstream of oncogenic Akt signaling in thymocytes were shown to result from EIF4E hyper activation, through a process that is specific to the 4EBP1 arm of oncogenic mTOR but that does not rely on rpS6 phosphorylation [38]. More potent inhibition of mTORC1 might thus impact on Mcl-1 expression in BT474 cells. We cannot rule out, moreover, the involvement of mechanisms capable of enhancing the stability of the Mcl-1 protein, such as the one that relies on the deubiquitinating enzyme USP9X [39], which is also involved in HER2 stability [40]. The resistance of Mcl-1 expression to mTORC1 inhibition by compounds that are used in the clinic revealed here, suggests that strategies aiming at inhibiting Mcl-1 transcription or at inhibiting the protein itself might constitute a more efficient, and reliable, approach than these that target its translation.

RAD001 treatment of BT474 cells not only leaves cell viability unaltered, but it protects cells against death induced by Mcl-1 depletion. Thus, active, RAD001 sensitive dependent death signals are involved in installing Mcl-1 dependence. It has been established, over the last decade, that the pro-apoptotic multidomain proteins Bax and Bak play a major role in the apoptotic response of mammalian cells. Moreover, numerous data have converged towards the notion that the BH3 domains of some "activator" BH3-only proteins (that of Bid, Bim and Puma) have the innate ability to interact with these proteins and to activate them. Thus, anti-apoptotic proteins allow cell survival by binding to their pro-apoptotic counterparts, thereby preventing a low affinity but high efficiency interaction between "activator" BH3-only proteins and multidomain proteins to occur and to kill cells. In support to this, we recently established that the ability of PUMA to activate Bax renders cells that constitutively express it dependent upon the sustained BH3-binding activity of Bcl-2 and Bcl-xL for survival [15]. Our observations that cell death rates induced by Mcl-1 depletion in BT474 cells are decreased by the co depletion of Bim are also mostly consistent with this view. Numerous studies (in particular in lung and breast cancer cells, [41]) have hinted on a role of the Bim/Mcl-1 balance in the control of survival, but very few have shown, as it is the case here, that the mechanism involved relies on Mcl-1 counteracting the ability of Bim to promote cell death, rather than the ability of Bim to erode the cytoprotective effect of Mcl-1.

It rises from above that signaling pathways that lead to the expression and the stability of Bim will actively contribute to render Mcl-1 expression required for survival. Our finding that Bim expression can be detected in lysates that were prepared from 5 HER2-amplified tumors that had received no treatment indicate that such pathways are active in this malignancy. Mechanisms that regulate Bim transcription in particular might be effective, as suggested by the possible enrichment for some Bim transcripts in HER2 amplified tumors revealed by our investigation of publicly available expression data from breast cancer. Our finding that RAD001 negatively regulates Bim expression indicate that mTORC1, which plays an important oncogenic role in HER2 amplified tumors, might contribute to this expression. The pro-apoptotic role our data attribute to the mTOR pathway is somewhat reminiscent to that reported for its downstream kinase S6K in hepatocytes, where S6K contributes to Bim expression [42]. Our data suggest that mTORC1 favors Bim expression by controlling the expression and the activity of c-Myc, and that this transcription factor is involved is the constitutive expression of Bim in BT474 cells. The results of our ChIP assays indicate that RAD001 sensitive c-Myc might be directly involved in the transcription of Bim in BT474 cells. As the mTOR pathway is frequently active in HER2 overexpressing breast cancers and regulates c-Myc activity, our results imply that the corresponding tumor cells might frequently express constitutive Bim. This constitute a molecular vulnerability that renders the sustained anti-apoptotic activity of Mcl-1 necessary for survival. Thus, one promising approach for the treatment of HER2 overexpressing breast cancers might be one that relies on the use of inhibitors of the anti-apoptotic activity of Mcl-1.

## Conclusions

Our work provides strong support to the notion that some tumor cells might depend upon a limited number of anti-apoptotic Bcl-2 like proteins (Bcl-2L) for their survival. It establishes that this "Bcl-2L dependence" extends to HER2 amplified tumors, and that, in these tumors, it relies, at least in part, on the interconnected pathways that lead to pro-apoptotic Bim and anti-apoptotic Mcl-1 expressions. This implies that current targeted approaches need to influence the balance between Bim and Mcl-1 to efficiently affect cancer cell survival. It also implies that novel strategies (such as these based on the use of Bcl-2 homologues inhibitors) that directly act upon this balance without interfering with the rest of the HER2 network are a promising alternative for the treatment of this disease.

## List of abbreviations

ChIP: Chromatin Immunoprecipitation; CIC: cancer initiating cells; FBS: fetal bovine serum; HER: human epidermal growth factor receptor; PBS: Phosphate Buffer Saline; SDS: Sodium Dodecyl Sulfate; siRNA: small interfering RNA; TBS-T: Tris Buffered Saline Tween; TSE: Triton SDS EDTA Buffer; UTR: untranslated region

## Competing interests statement

The authors declare that they have no competing interests.

## Authors' contributions

MC conceived of the study, participated in its design and helped to draft the manuscript. BN carried out biochemical and cell biology assays, analyzed data and participated in the design of the study. CC, MG and YG carried out biochemical and cell biology assays. FG prepared tumor lysates and performed biochemical assays. WG and CC carried out in silico assays and performed statistical analysis. LC participated in the design of the study and performed statistical analysis. PJ participated in the design of the study and coordinated the in silico and statistical assays. FB carried out biochemical assays and contributed to the design of the study. BB participated in the design of the study, carried out the ChIP assays and helped to draft the manuscript. OC participated in the design of the study and helped to draft the manuscript. SBN participated in the design of the study, helped to draft the manuscript and carried out the mammosphere assays. PJ conceived of the study, participated in its design, coordinated the studies and wrote the manuscript. All authors read and approved the final manuscript.

## Supplementary Material

Additional file 1 **Bim dependent induction of cell death by Mcl-1 knock down in BT474 cells**. Top panel. BT474 cells were transfected with the indicated siRNA as described in Figure 1 and cell death was evaluated by a trypan blue procedure. Data are mean ± se of three independent experiments. Middle panel. BT474 cells were transfected with control siRNA, Bim siRNA, PUMA siRNA and/or Mcl-1 siRNA as indicated and Annexin V expression was analyzed 48 hours later. Data are mean ± se of three independent experiments. Bottom panel Western blot analysis was performed to confirm Mcl-1 down regulation in cells transfected with Mcl-1 siRNA together with control (ctr), Bim or Puma siRNA. Click here for file

Additional file 2 **Down regulation of Mcl-1 by siRNA transfection of MCF7 and SKBR3 cells**. The indicated cells were transfected with the indicated siRNA and western blot analysis of Bim and Mcl-1 was performed 48 hours later using tubulin as a loading control. Click here for file

Additional file 3 **Expression of Mcl-1 and of Bim in BT474, SKBR3, MCF7 and MCF10A cells**. Western blot analysis was performed using lysates from the indicated cells. Click here for file

Additional file 4 **Lack of effect of control siRNA transfection on the expression of Mcl-1, Bim, Noxa and Puma in BT474 cells**. BT474 cells were left untreated or transfected with a control siRNA and western blot analysis was performed 48 hours later, using tubulin as a loading control. Click here for file
