# Supplementary material for: c-Myc dependent expression of pro-apoptotic Bim renders HER2-overexpressing breast cancer cells dependent on anti-apoptotic Mcl-1
Source: Mol Cancer. 2011 Sep 7;10:110. doi: 10.1186/1476-4598-10-110 (PMC3175201; doi:10.1186/1476-4598-10-110)

Trypan blue positive cells (%)

**siCtr.**	**4% ± 0.007%**
**siMcl-1**	**29% ± 0.01%**

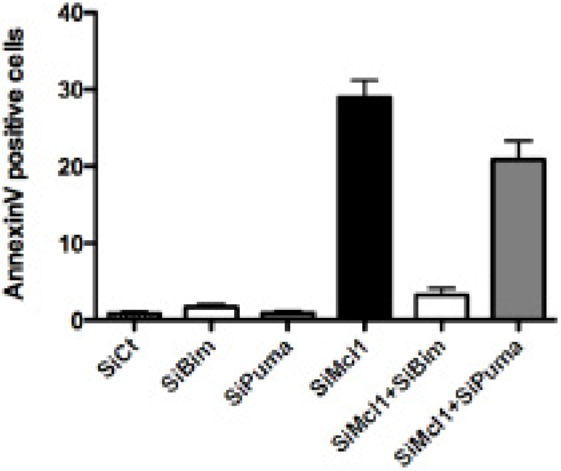

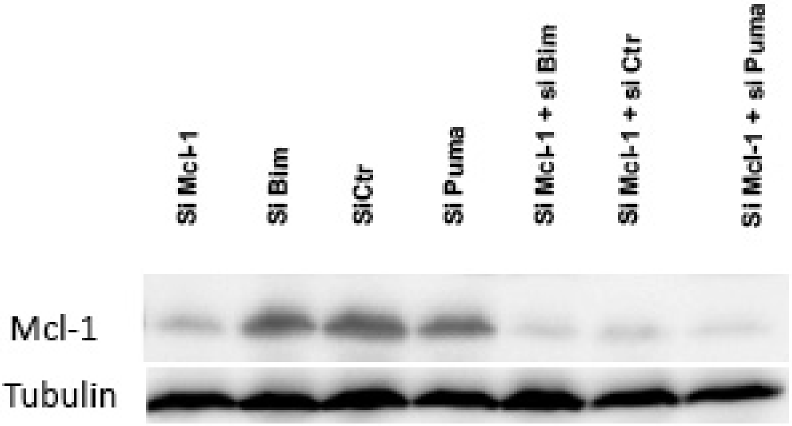

Supplement: Additional file 1 — Bim dependent induction of cell death by Mcl-1 knock down in BT474 cells. Top panel. BT474 cells were transfected with the indicated siRNA as described in Figure 1 and cell death was evaluated by a trypan blue procedure. Data are mean ± se of three independent experiments. Middle panel. BT474 cells were transfected with control siRNA, Bim siRNA, PUMA siRNA and/or Mcl-1 siRNA as indicated and Annexin V expression was analyzed 48 hours later. Data are mean ± se of three independent experiments. Bottom panel Western blot analysis was performed to confirm Mcl-1 down regulation in cells transfected with Mcl-1 siRNA together with control (ctr), Bim or Puma siRNA. [file 1476-4598-10-110-S1.PDF]
