# Supplementary figures and images for: c-Myc dependent expression of pro-apoptotic Bim renders HER2-overexpressing breast cancer cells dependent on anti-apoptotic Mcl-1
Source: Mol Cancer. 2011 Sep 7;10:110. doi: 10.1186/1476-4598-10-110 (PMC3175201; doi:10.1186/1476-4598-10-110)

MCF7

SKBR3

si ctr

si Mcl-1

si ctr

si Mcl-1

Mcl-1

Bim(EL)

Tubulin

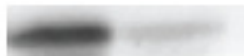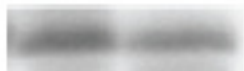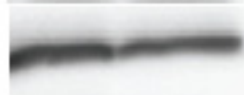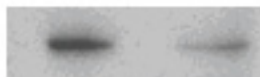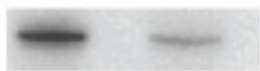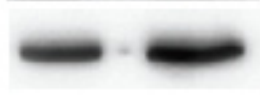

Supplement: Additional file 2 — Down regulation of Mcl-1 by siRNA transfection of MCF7 and SKBR3 cells. The indicated cells were transfected with the indicated siRNA and western blot analysis of Bim and Mcl-1 was performed 48 hours later using tubulin as a loading control. [file 1476-4598-10-110-S2.PDF]

BT474

MCF10A

SKBR3

MCF7

Mcl1

Bim

Tubulin

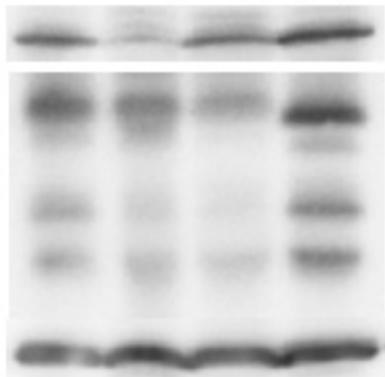

Supplement: Additional file 3 — Expression of Mcl-1 and of Bim in BT474, SKBR3, MCF7 and MCF10A cells. Western blot analysis was performed using lysates from the indicated cells. [file 1476-4598-10-110-S3.PDF]

Unt.

Si cont

Mcl-1

Bim

Noxa

Puma

Tubulin

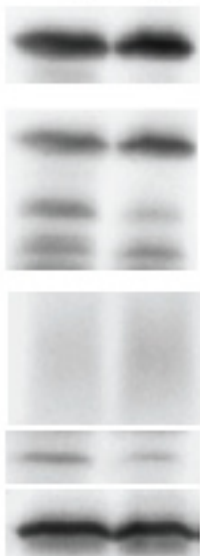

Supplement: Additional file 4 — Lack of effect of control siRNA transfection on the expression of Mcl-1, Bim, Noxa and Puma in BT474 cells. BT474 cells were left untreated or transfected with a control siRNA and western blot analysis was performed 48 hours later, using tubulin as a loading control. [file 1476-4598-10-110-S4.PDF]
